# Single-center study for robotic-assisted laparoscopic sacropexies: a one-fits-all strategy for pelvic organ prolapse?

**DOI:** 10.1007/s00404-022-06735-6

**Published:** 2022-08-16

**Authors:** Pawel Mach, Cara Kaufold, Peter Rusch, Rainer Kimmig, Paul Buderath

**Affiliations:** Department of Obstetrics and Gynecology, University Hospital of Essen, Essen, Germany

**Keywords:** Sacrocolpopexy, Cervicosacropexy, Robotic-assisted surgery, POP

## Abstract

**Purpose:**

Sarcopenia has been established as the “gold standard” for the treatment of pelvic organ prolapse (POP). Minimal invasive laparoscopy can help to reduce the risks of open access surgery. We compare the surgical results and outcomes of robotic-assisted sacropexies.

**Methods:**

In this monocentric retrospective study we enrolled 49 patients operated on symptomatic POP. Patients were divided into two groups according to the type of robotic-assisted sacropexy: patients with a history of hysterectomy received robotic-assisted sacrocolpopexy (RSCP; *n* = 19), while patients with subtotal hysterectomy received robotic-assisted cervicosacropexy (RCSP; *n* = 30). Failure was defined as recurrence of the disease with a need for reoperation. Validated questionnaires (the Pelvic Floor Distress Inventory—20 (PFDI-20) and Pelvic Floor Impact Questionnaire—7 (PFIQ-7)), were used for evaluation of patients quality of life postoperatively.

**Results:**

The comparison between RCSP versus RSCP showed that the latter is related to slightly but not significantly increased recurrence rates and a higher impact of POP symptoms on quality of life in long-term follow-up (*p* = 0.04). Perioperative data showed similar complication rates in both RSP types but shorter postoperative time of bladder catheterization in the case of RCSP (*p* = 0.008).

**Conclusions:**

The monocentric long-term data confirm that RSP is a safe and effective method of surgical POP treatment, regardless of the site of the anatomical compartment. In comparison to RSCP, RCSP is associated with a lower impact of POP symptoms on patients’ quality of life with a tendency to slightly lower rates of POP recurrence.

## What does this study add to the clinical work

**Table:** 

The monocentric long-term data confirm that RSP is a safe and effective method of surgical POP treatment, regardless of the site of the anatomical compartment. In comparison to RSCP, RCSP is associated with a lower impact of POP symptoms on patients’ quality of life with a tendency to slightly lower rates of POP recurrence.	

## Purpose

Age and obesity are main risk factors for pelvic organ prolapse (POP). Due to longer life expectancy among women, there is increasing incidence of POP, and the lifetime risk for surgical treatment is 13–20% [1]. Treatment of POP includes surgical and conservative measures and depends on the location of the defect.

Sacropexie (SP) has been established as the “gold standard” for the treatment of prolaps of the vaginal apex (with or without cervix/uterus), but there is often a predisposition to anterior or posterior defects, which should also be included in the surgical strategy for POP repair. Compared to other invasive measures, such as vaginal approaches, SP is associated with better surgical outcomes and lower complication rates [2].

SP has traditionally been performed by laparotomy, but minimally invasive methods show comparable surgical outcomes with fewer complications, less pain, and faster convalescence [3]. Long-term results of laparoscopic SC have shown a rate of reoperation for recurrence of 3.3% after 7 years of follow-up [4]. Due to the proximity of critical anatomical structures and the limited access to the operating field, the minimally invasive procedure requires experienced surgeons with good laparoscopic skills. These factors result in a longer operating time and a longer learning curve compared to open surgery [5].

In 2005, Intuitive^®^ Surgery Inc. (Intuitive^®^ Surgical Inc., Sunnyvale, California, US) entered the field of minimally invasive gynecological surgery with the FDA approval for the first DaVinci^®^ robotic-system. Robotic surgery is known for improved ergonomics for the surgeon and flexible surgery in anatomy with limited access, e.g., female pelvic spaces such as dissection of posterior vaginal wall down to the level of the levator ani. Studies have demonstrated a short learning curve for robotic-assisted gynecological surgery compared to conventional laparoscopy [6], making the device ideal for the complex tasks of minimally invasive pelvic floor repair. Robotic-assisted sacropexy (RSP) tends to become a new standard for many surgeons [7].

The main goal of POP surgery is the improvement of quality of life rather than simple anatomical restoration [8]. Thus, POP treatment is complex and must consider multiple surgical and non-surgical outcome parameters besides with direct and indirect effects on quality of life, such as urinary continence and body image.

On the other hand, surgical options of POP repair may be limited for patients with relevant comorbidities and risk factors. Age and obesity are known risk factors for surgery, especially for open access. Thus, minimal invasive laparoscopy can help to reduce the risks of open access surgery for the patient, while robotic assistance could additionally help to overcome the limited availability of only few highly skilled straight-stick laparoscopists in the context of complex pelvic floor surgery [9, 10]. Initial experiences are promising, but SP remains the gold standard.

In this monocentric retrospective clinical cohort-study, we analyzed the feasibility of RSP as the primary objective in the context of surgical pelvic floor repair. We compare the surgical results and outcomes of RSP following prior hysterectomy (robotic assisted sacrocolpopexy, RSCP) versus RSP with subtotal, i.e., supracervical hysterectomy (robotic assisted cervicosacropexy, RCSP). Our focus lies on the intra- and perioperative course of treatment, patient-related outcome parameters, and anatomical restoration. We discuss whether robotic assistance helps to establish RSP as a “one-fits-all” concept in the context of POP surgery, irrespective of involved anatomical compartment (apical, anterior, posterior, combined defect) and of patient-related comorbidities.

## Methods

### Patient characteristics

This retrospective study enrolled consecutively 49 patients with a mean age of 63.5 years (standard deviation (SD) 10.7) who were treated in the Department of Gynecology and Obstetrics at the University Hospital of Essen, Germany, between 2012 and 2019. All patients were operated on due to symptomatic POP stage II or greater uterine/vaginal vault prolapse in the department. Diagnosis of POP was based on clinical examination prior to the surgery. The clinical stage of POP was determined according to the Pelvic Organ Prolapse Quantification (POP-Q) System [11].

The information recorded at the time of surgery included age, body mass index, parity, prior POP or anti-incontinence surgery, prior hysterectomy, estimated blood loss, operative time, perioperative complications and length of hospital stay, which were retrospectively obtained from patient medical records. Ethical approval was granted by the local ethics committee of the University of Duisburg-Essen (20-9354-BO). Patients were divided into two groups according to the type of RSP: patients with a history of hysterectomy received RSCP (*n* = 19), while patients with subtotal hysterectomy received RCSP (*n* = 30).

### Surgical procedure

All surgical procedures were performed with a DaVinci^®^ Si robotic system (Intuitive^®^ Surgical Inc., Sunnyvale, California, US) between 2012 and 2015 and with DaVinci^®^ Xi robotic system from 2015 to 2019. All procedures were performed by a single surgeon using the same surgical technique and the same surgical materials in a standardized procedure:

The patients are placed in a dorsal lithotomy and steep Trendelenburg position at approximately 30°. The Veress needle is used for insufflation through the umbilicus to generate pneumoperitoneum to an intra-abdominal pressure of 15 mmHg. A 8 mm umbilical trocar is placed. Three 8 mm robotic trocars are placed bilaterally in horizontal configuration under direct vision. Ports are placed usually on the level of umbilicus; however, it may vary depending on characteristics of the patient. One assistant 10 mm port is placed 3 cm medial from the anterior superior iliac spine, lateral to the rectus abdominis muscles. Lateral to the promontory, between the mesorectum and the iliac vessels, the peritoneum is opened medial to the ureter. The avascular space is developed to the right sacral ligaments at the S1–S2 level. The peritoneum is opened or tunneled along the course of the sacrouterin ligament to approach the cervix/vagina. Preparation of the front vaginal wall is done to just above the trigonum vesicae, about 5 cm distal to the vaginal tip and the posterior vaginal wall up to the perineum. Then, supracervical hysterectomy is performed if needed.

Y-shaped mesh (DynaMesh, FEG Textile Technology Research and Development Company, Aachen Germany; 5 cm leg width or 3 cm width, polyvinylidene fluoride [PVDF]) is placed on the posterior and anterior vaginal wall and attached with an absorbable suture (VICRYL 2–0, Johnson & Johnson, New Brunswick, NJ, USA). The front and posterior leg of the mesh are attached to the cervix or fixed on the vaginal wall. The Y-shaped mesh is fixed at the level of anterior longitudinal ligaments S1 or S2 using 2 non-absorbable sutures (ETHIBOND 0, Ethicon, Raritan, NJ, USA). Subsequently, the peritoneum over the mesh is closed again using absorbable sutures. Finally, an intraoperative clinical examination is carried out to ensure that the mesh is in a tension-free position. No surgical procedures for stress urinary incontinence are performed simultaneously. The postoperative examination was performed by the surgeon himself.

### Outcomes

Patient outcome was evaluated according to symptoms, while failure was defined as recurrence of the disease with a need for reoperation. Validated questionnaires (the Pelvic Floor Distress Inventory—20 (PFDI-20) and Pelvic Floor Impact Questionnaire—7 (PFIQ-7)), were used for evaluation of patients reported outcomes. Their primary objective was the surgical effect on symptoms of pelvic floor dysfunction and the impact of symptoms on quality of life.

The PFDI-20 questionnaire is a disease-specific set of 20 questions and consists of three scales: the Pelvic Organ Prolapse Distress Inventory (POPDI-6), Colorectal–Anal Distress Inventory (CRADI-8), and Urinary Distress Inventory (UDI-6). The possible responses are 0 (not present), 1 (not at all), 2 (somewhat), 3 (moderately), and 4 (quite a bit). Higher scores indicate more symptom distress [12].

The PFIQ-7 questionnaire consists of 21 questions and assesses the extent to which bladder, bowel, or vaginal symptoms affect activities, relationships, and feelings. The PFIQ-7 consists of three scales: the Urinary Impact Questionnaire (UIQ-7), the Colorectal–Anal Impact Questionnaire (CRAIQ-7), and the Pelvic Organ Prolapse Impact Questionnaire (POPIQ-7). The possible responses are 0 (not at all), 1 (somewhat), 2 (moderately), and 3 (quite a bit). Higher scores indicate more impact of POP symptoms on daily activity [12].

### Statistical analysis

The distribution of variables was checked with the Shapiro–Wilk test. Characteristics of the study population were reported as either means with SDs or medians with interquartile ranges (IQRs). Differences in discrete variables were analyzed using the Kruskal–Wallis test and student’s *t* test, and frequency counts were analyzed using the chi-squared test. Logistic regression analysis was used to calculate odds ratios (ORs) and 95% confidence intervals (95% CIs). A *p* value of < 0.05 was considered indicative of statistical significance. All analyses were performed using MedCalc version 17.9.7 (MedCalc Software bvba, Ostend, Belgium).

## Results

There were 49 women who received RSP Mean age was 63.5 years (SD 10.7), mean BMI was 24.7 kg/m^2^ (SD 3.8). The mean follow-up time was 56.2 months (SD 26.5), the median parity was 2 (IQR 1–2), the median number of previous vaginal deliveries was 2 (IQR 1–2), and the median number of previous cesarean sections was 0 (IQR 0). There were 12 (24.4%) patients who were using tobacco, and the median ASA classification grade was 2 (IQR 2–3).

9 (18.4%) patients had a previous POP repair, 21 (42.8%) patients had symptoms of urinary incontinence preoperatively, 5 (10.2%) patients were diagnosed in POP-Q stage II, 33 (67.3%) patients were in POP-Q stage III, and 11 (22.4%) were in stage IV. All patients suffered from vaginal bulge. 2 (4.1%) patients suffered from apical prolapse only, 28 (57.1%) had apical and anterior prolapse, 3 (6.1%) had apical and posterior prolapse, and 16 (32.7%) patients suffered from prolapse in all three compartments. Preoperative patient characteristics in both groups are presented in Table 1.

**Table 1:** Patients characteristics of RSP

	RSCP (robotic assisted sacrocolpopexy; *n* = 19)	RCSP (robotic assisted cervicosacropexy; *n* = 30)	*p* value
Age at surgery, years, mean (SD)	65.4 (8.8)	62.3 (11.7)	0.3
BMI, kg/m^2^, mean (SD)	24.8 (3.9)	24.6 (3.9)	0.8
Parity, median (IQR)	2 (2–1)	2 (2–2)	0.3
Previuos vaginal deliveries, *n* (%)	2 (1–2)	2 (1.2–2)	0.3
Previous cesarean section, *n* (%)	1 (5.2)	3 (10)	0.52
Smoker, *n* (%)	6 (31.6)	6 (20)	0.28
ASA classification grade, median (IQR)	2 (2–3)	2 (2–3)	0,37
Previous prolapse repair, *n* (%)	4 (21)	5 (16.6)	0.93
Urinary incontinence symptoms, *n* (%)	8 (42)	13 (43.3)	0.96
POP-Q stage preoperative, *n* (%)			0.86
II	2 (10.5)	3 (10)	
III	12 (63.1)	21 (70)	
IV	5 (26.3)	6 (20)	
Location of prolapse, *n* (%)			0.87
Apical only	1 (5.3)	1 (3.3)	
Apical and anterior	12 (63.2)	16 (53.3)	
Apical and posterior	1 (5.3)	2 (6.6)	
Apical, anterior and posterior	5 (26.2)	11 (36.6)	

*ASA* American Society of Anesthesiologist physical status classification; *BMI* body mass index

The mean operative time was 197 min (SD 55.5) and the operative time did not change during the study. 17 (34.7%) patients had intraoperative lysis of adhesions prior to RSP. The median estimated blood loss was 104 mL (IQR 60–193). No intraoperative complications were observed in our cohort. There was no case of conversion to open surgery. Postoperative catheter remained in place for a mean of 1.7 (SD 1.3) days after surgery.

Postoperative complications were reported in 8 (16.3%) patients, of which 3 (6.1%) developed urinary tract infection, 2 (4.1%) patients had mesh erosion, and 3 (6.1%) patients had urinary retention. Using the Clavien–Dindo classification, 6 (12.2%) patients were classified as grade I, and 2 (4.1%) patients were grade II. Post-operative complications were associated with surgery if occurring within 30 days after RSP. There were no reports of de novo stress incontinence after surgery. 3 (6.1%) patients suffered from recurrence of prolapse, and reoperation due to symptoms was required. The surgical outcomes of RSCP and RCSP are presented in Table 2.

**Table 2:** Surgical outcome of RSCP and RCSP

	RSCP (robotic assisted sacrocolpopexy; *n* = 19)	RCSP (robotic assisted cervicosacropexy; *n* = 30)	*p* value
Estimated blood loss, mL, median (IQR)	138 (25–166)	88 (80–191)	0.19
Operative time, minutes, mean (SD)	209.6 (65)	189.4 (47.8)	0.22
Lysis of adhesions, *n* (%)	12 (40)	5 (26.3)	0.33
Hospital stay, days, median (IQR)	6 (5–7)	5 (4–6)	0.25
Postoperative catheter placement, days, mean (SD)	2,4 (1,8)	1,4 (0,5)	0,008
Follow-up duration, months, mean (SD)	44.5 (14.8)	62.6 (29.6)	0.07
Prolapse recurrence, *n* (%)	2 (10.5)	1 (3.3)	0.31
Complications, *n* (%)	4 (21)	4 (13)	0.4
Postoperative complications using Clavien–Dindo Classification, *n* (%)			0.4
Grade I	3 (15.7)	3 (10)	
Grade II	1 (5.2)	1 (3.3)	

No patient-related factors were associated with the risk of recurrence, including parity, BMI, age, tobacco use, preoperative POP-Q stage, and prior descensus surgery. The results of the logistic regression models of prolapse recurrence are presented in Table 3. 33 out of 49 (67.3%) patients completed the PFDI-20 and PFIQ-7 questionnaires. The overall medianPFDI-20 score was 33.3 (IQR 15.6–61.9), that of POPDI-6 was 6.2 (IQR 0–12.5), that of CRAD-8 was 8.6 (IQR 3.1–18.7), and that of UDI-6 was 10.4 (IQR 4.1–37.5). The overall median PFIQ-7 score was 33.3 (IQR 15.6–61.9), that of PFIQ-7 was 4.7 (0–28.5), that of UIQ-7 was 4.7 (0–19), that of CRAIQ-7 was 0 (0–1.2), and that of POPIQ-7 was 0 (0). Quality of life follow-up outcomes in both RSP groups are shown in Table 4.

**Table 3:** Logistic regression of prolapse recurrence

	OR	95% CI
BMI	0.92	0.64–1.33
Supracervical hysterectomy	0.31	0.02–3.87
Age	0.97	0.87–1.09
Smoker	1.31	0.1–16.55
POP-Q stage preoperative	3.53	0.35–35.92

**Table 4:** Quality of life measures

	RSCP (robotic assisted sacrocolpopexy; *n* = 19)	RCSP (robotic assisted cervicosacropexy; *n* = 30)	*p* value
Pelvic Floor Distress Inventory—20 (PFDI-20), median (IQR)	44.8 (19.7–130.9)	31.2 (14.8–48.9)	0.2
POPDI-6, median (IQR)	8.3 (0–18.7)	4.2 (0–12.5)	0.31
CRAD-8, median (IQR)	6.2 (0.7–23.4)	9.4 (3.1–14.8)	0.8
UDI-6, median (IQR)	29.2 (4.2–52.1)	8.3 (4.2–33.3)	0.62
Pelvic Floor Impact Questionnaire—7 (PFIQ-7), median (IQR)	23.8 (4.8–66.7)	4.7 (0–13.1)	0.04
UIQ-7, median (IQR)	9.5 (0–28.6)	4.8 (0–13.1)	0.49
CRAIQ-7, median (IQR)	0 (0–14.3)	0 (0)	0.1
POPIQ-7, median (IQR)	0 (0–9.5)	0 (0)	0.05

## Discussion

Our data confirm that RSP is an effective and safe method for prolapse repair, regardless of the site of the anatomical damage and comorbidities linked to risk factors for POP. The comparison between RCSP versus RSCP showed that the latter is related to slightly but not significantly increased recurrence rates and a higher impact of POP symptoms on quality of life in long-term follow-up. Perioperative data showed similar complication rates in both RSP types but shorter postoperative time of bladder catheterization in the case of RCSP.

The recurrence rate for all patients was 6.1% over a mean follow-up time of 4.5 years. A retrospective study with a long follow-up of 7 years revealed that RSP shows sustained success in the treatment of symptomatic POP [13]. Other studies with a long-term follow-up of more than 5 years with cure rates of 93.3–100% showed low complication rates and good patient acceptance [14].

To date, there are no randomized trials with long-term RSP outcome data. There are four randomized controlled trials comparing RSC with laparoscopic SP with the longest median follow-up of 25 months [15–18]. Healing rates for all compartments were 85–100%, no recurrences were observed, and no reoperations were required [15–18]. In comparison to our results, these data suggest that recurrences observed after RSP may occur at a later point in time after surgery.

We did not observe significant differences when comparing recurrence rates between RSCP and RCSP. However, in the group receiving RCSP, the recurrence rate was only a third of that in the RSCP group. This seems plausible taking into account the effect of prior surgery on the integrity of tissue and the presence of adhesions. Moreover, the uterine cervix represents a more firm structure to fix the mesh upon compared to the apical vagina. Our results are similar to those of a prospective study by van Zanten et al. [19]. After 12 months of follow-up, 91% of their patients who had RSCP, and 99% of those who had supracervical hysterectomy with RCSP showed anatomical success of the apical compartment, although the average operative time was 38 min longer [19]. Another study showed similar results, suggesting a longer operative time but fewer complications in women undergoing RSP and hysterectomy during the same procedure compared to patients undergoing RSCP only [20].

The reported median surgery time of RSP varies significantly between studies [21, 22]. In a recent meta-analysis with a total of 3014 patients, the median operating time for RSP was 226 min [23]. Another prospective study observed an operating time for RSP of 125 min (90–270 min) [24]. In our study, the mean operative time for the whole study population was 197 min, which is similar to previous reports.

Dubinskaya et al. observed significantly increased operative time by 17.8 min when performing concomitant hysterectomy compared to RSCP alone. Although we did not observe any significant differences, our results show another tendency as the mean operative time was increased by 20 min when performing RSCP. This difference may be due to time-consuming preparation of the bladder in patients with a history of hysterectomy and potentially more adhesions after the previous surgery.

The subjective outcomes were assessed using the validated PFDI-20 and PFIQ-7 questionnaires. We found no differences in symptoms, but the impact of POP symptoms on patients’ quality of life was lower in the group of patients undergoing RCSP. We did not assess the PFDI-20 and PFIQ-7 questionnaires before operation, which could be useful in determining changes in symptom severity over time. Nevertheless, Karjalainen et al. showed that a Patient Acceptable Symptom State (PASS) score of ≤ 60 for the PFDI-20 scale represents a limit at which patients consider themselves well [25]. In our study, in both groups, the median postoperative PFDI-20 score was under 60, meaning the symptom state was acceptable, regardless of the type of RSP. Similar results are reported in a long-term evaluation of subjective outcomes of RSP [26].

Postoperative complications occurred in 16% of patients and did not differ among both groups of RSP. Mesh erosion occurred in 4%, which required reoperation. A systematic review of RSP showed comparable mesh erosion rates in the range of 0–8% [27]. No suture erosion occurred in our study, which may be due to precise placement of sutures in the vaginal wall. We did not observe any bladder or ureter injuries, but three patients had postoperative pathological urine retention, which required an operative correction. In two cases we performed partial mesh removal. In one case we removed the sutures of the caudal suture line due to elevated tension and potential distortion of the ureteral ostium. The operations were performed robotic- and cystoscopic-assisted. In randomized controlled trials, bladder injuries were reported in 0–6% cases [15–18].

Our mean follow-up duration was 3.7–5.2 years after surgery. Long-term follow-up data for RSP indicate reoperation rates of 2.7–3.3% [3]. Our data are consistent with this, showing a rate of 3.3% in the RCSP group. Nevertheless, this study has limitations as a single-center retrospective study. Furthermore, the number of patients in both groups is small which may be a reason for the lack of statistical significance for the observed differences. Finally, we did not assess subjective outcomes preoperatively, which is a fundamental tool when assessing the effectiveness of treatments. Because of that, it is important to design and perform prospective randomized trials of RSP.

## Conclusions

The monocentric long-term data confirm that RSP is a safe and effective method of surgical POP treatment, regardless of the site of the anatomical compartment. In comparison to RSCP, RCSP is associated with a lower impact of POP symptoms on patients’ quality of life with a tendency to slightly lower rates of POP recurrence.

## Data Availability

All data are available from pawel.mach@uk-essen.de.

## References

[1] Wu JM, Matthews CA, Conover MM (2014). Lifetime risk of stress urinary incontinence or pelvic organ prolapse surgery. Obstet Gynecol.

[2] Wang J, Wang X, Hua K, Chen Y (2019). Laparoscopic sacrocolpopexy plus colporrhaphy with a small intestine submucosa graft versus total pelvic floor reconstruction for advanced prolapse: a retrospective cohort study. Int Neurourol J.

[3] Linder BJ, Occhino JA, Habermann EB (2018). A national contemporary analysis of perioperative outcomes of open versus minimally invasive sacrocolpopexy. J Urol.

[4] Pacquée S, Nawapun K, Claerhout F (2019). Long-term assessment of a prospective cohort of patients undergoing laparoscopic sacrocolpopexy. Obstet Gynecol.

[5] Ganatra AM, Rozet F, Sanchez-Salas R (2009). The current status of laparoscopic sacrocolpopexy: a review. Eur Urol.

[6] Lenihan JPJ, Kovanda C, Seshadri-Kreaden U (2008). What is the learning curve for robotic assisted gynecologic surgery?. J Minim Invasive Gynecol.

[7] Carroll AW, Lamb E, Hill AJ (2012). Surgical management of apical pelvic support defects: the impact of robotic technology. Int Urogynecol J.

[8] Srikrishna S, Robinson D, Cardozo L (2010). A longitudinal study of patient and surgeon goal achievement 2 years after surgery following pelvic floor dysfunction surgery. BJOG.

[9] Kale A, Biler A, Terzi H (2017). Laparoscopic pectopexy: initial experience of single center with a new technique for apical prolapse surgery. Int Braz J Urol.

[10] Banerjee C, Noé KG (2011). Laparoscopic pectopexy: a new technique of prolapse surgery for obese patients. Arch Gynecol Obstet.

[11] Bump RC, Mattiasson A, Bø K (1996). The standardization of terminology of female pelvic organ prolapse and pelvic floor dysfunction. Am J Obstet Gynecol.

[12] Barber MD, Walters MD, Bump RC (2005). Short forms of two condition-specific quality-of-life questionnaires for women with pelvic floor disorders (PFDI-20 and PFIQ-7). Am J Obstet Gynecol.

[13] Jong K, Klein T, Zimmern PE (2018). Long-term outcomes of robotic mesh sacrocolpopexy. J Robot Surg.

[14] Shimko MS, Umbreit EC, Chow GK, Elliott DS (2011). Long-term outcomes of robotic-assisted laparoscopic sacrocolpopexy with a minimum of three years follow-up. J Robot Surg.

[15] Anger JT, Mueller ER, Tarnay C (2014). Robotic compared with laparoscopic sacrocolpopexy: a randomized controlled trial. Obstet Gynecol.

[16] Kenton K, Mueller ER, Tarney C (2016). One-year outcomes after minimally invasive sacrocolpopexy. Female Pelvic Med Reconstr Surg.

[17] Paraiso MFR, Jelovsek JE, Frick A (2011). Laparoscopic compared with robotic sacrocolpopexy for vaginal prolapse: a randomized controlled trial. Obstet Gynecol.

[18] Illiano E, Ditonno P, Giannitsas K (2019). Robot-assisted Vs laparoscopic sacrocolpopexy for high-stage pelvic organ prolapse: a prospective, randomized, single-center study. Urology.

[19] van Zanten F, Schraffordt Koops SE, O’Sullivan OE (2019). Robot-assisted surgery for the management of apical prolapse: a bi-centre prospective cohort study. BJOG.

[20] Dubinskaya A, Hernandez-Aranda D, Wakefield DB, Shepherd JP (2020). Comparing laparoscopic and robotic sacrocolpopexy surgical outcomes with prior versus concomitant hysterectomy. Int Urogynecol J.

[21] Di Marco DS, Chow GK, Gettman MT, Elliott DS (2004). Robotic-assisted laparoscopic sacrocolpopexy for treatment of vaginal vault prolapse. Urology.

[22] Salamon CG, Culligan PJ (2013). Subjective and objective outcomes 1 year after robotic-assisted laparoscopic sacrocolpopexy. J Robot Surg.

[23] Yang J, He Y, Zhang X (2021). Robotic and laparoscopic sacrocolpopexy for pelvic organ prolapse: a systematic review and meta-analysis. Ann Transl Med.

[24] Seror J, Yates DR, Seringe E (2012). Prospective comparison of short-term functional outcomes obtained after pure laparoscopic and robot-assisted laparoscopic sacrocolpopexy. World J Urol.

[25] Karjalainen PK, Mattsson NK, Jalkanen JT (2021). Minimal important difference and patient acceptable symptom state for PFDI-20 and POPDI-6 in POP surgery. Int Urogynecol J.

[26] Linder BJ, Chow GK, Elliott DS (2015). Long-term quality of life outcomes and retreatment rates after robotic sacrocolpopexy. Int J Urol Off J Japanese Urol Assoc.

[27] Serati M, Bogani G, Sorice P (2014). Robot-assisted sacrocolpopexy for pelvic organ prolapse: a systematic review and meta-analysis of comparative studies. Eur Urol.

